# Activation of Sonic Hedgehog Leads to Survival Enhancement of Astrocytes via the GRP78-Dependent Pathway in Mice Infected with *Angiostrongylus cantonensis*

**DOI:** 10.1155/2015/674371

**Published:** 2015-04-16

**Authors:** Kuang-Yao Chen, Chien-Ju Cheng, Lian-Chen Wang

**Affiliations:** ^1^Graduate Institute of Biomedical Sciences, College of Medicine, Chang Gung University, Taoyuan 333, Taiwan; ^2^Department of Parasitology, College of Medicine, Chang Gung University, Taoyuan 333, Taiwan; ^3^Molecular Infectious Disease Research Center, Chang Gung Memorial Hospital, Taoyuan 333, Taiwan

## Abstract

*Angiostrongylus cantonensis* infection may cause elevation of ROS and antioxidants in the CSF of infected mice. Astrocytes may protect the surrounding neurons from oxidative stress-induced cell death by secreting Sonic hedgehog (Shh) via the PI3-K/AKT/Bcl-2 pathway. This study was conducted to determine the role of the Shh signaling pathway in *A. cantonensis*-infected BABL/c mice by coculturing astrocytes with living fifth-stage larvae or soluble antigens. The Shh pathway was activated with corresponding increases in the level of the Shh. Glial fibrillary acidic protein (GFAP) and Shh were increased in astrocyte cocultured with living fifth-stage larvae or soluble antigens. The survival of astrocytes pretreated with Shh was significantly elevated in cocultures with the antigens but reduced by its inhibitor cyclopamine. The expression of GRP78 and Bcl-2 was significantly higher in astrocytes pretreated with recombinant Shh. These findings suggest that the expression of Shh may inhibit cell death by activating Bcl-2 through a GRP78-dependent pathway.

## 1. Introduction

The Hedgehog (Hh) secreted proteins and signaling pathway play important roles in animal development [1, 2]. Sonic hedgehog (Shh), Desert hedgehog (Dhh), and Indian hedgehog (Ihh) are the three mammalian homologs that have been discovered. Ihh and Dhh are expressed in specific tissues, whereas Shh expression is ubiquitous. Moreover, Shh deficiency may lead to defects in the embryonic development of the neural tube, limbs, and foregut [3].

Shh signaling is mediated by a series of inhibitory steps. In the absence of Shh, the transmembrane Patched (Ptc) receptor blocks the function of the transmembrane protein Smoothened (Smo). After secretion of Shh, it binds to the Ptc receptor and Smo is activated. These changes initiate a signaling cascade that results in the activation of the transcription factor glioma-associated oncogene-1 (Gli1) [4]. Upon binding to Shh, Gli1 translocates into the nucleus and controls the transcription of the target genes* shh*,* ptc*,* bcl-2*, and* cyclin D*. These changes promote cellular proliferation, cellular survival, immune response, and cell fate determination in a variety of organs [5–7].

Activation of the Shh pathway has also been reported in neurological impairments. This phenomenon has been attributed to the protection of the central nervous system (CNS) through elevated expression of the antiapoptotic protein Bcl-2 [8, 9]. Moreover, Shh signaling may protect neurons from damage in Parkinsonism and Alzheimer's disease by regulating cell proliferation and apoptosis [10–13]. This signaling pathway also promotes blood-brain barrier (BBB) integrity and CNS immune responses. The role of the Shh pathway in endogenous immunity at the BBB protects the CNS against the entry of proinflammatory lymphocytes [14].

Astrocytes are the most abundant glial cells in the brain. These cells can reduce H_2_O_2_ toxicity by generation of antioxidants (glutathione peroxidase) and delay neuronal death under H_2_O_2_ generation [15]. During brain injury, the Shh pathway is activated in reactive astrocytes and plays a role in promoting cell proliferation [16, 17]. The secreted shh from activated astrocytes also protects cortical neurons against oxidative stress, suggesting a potential role for Shh in the treatment of brain ischemia and neurodegenerative disorders [18, 19].

Glucose-regulated protein 78 (GRP78), also called binding immunoglobulin protein (BiP), is an endoplasmic reticulum (ER) chaperone of the heat shock protein family [20, 21]. Under ER stress, activation of GRP78 may increase cell survival through the unfolded protein response [22]. In addition, induction of GRP78 may also protect cells from ER stress-induced apoptosis by activating Bcl-2 and inhibiting Bak, Bax, caspase, and CHOP [23, 24]. Under pathological damage conditions, this protein has protective effects on tissues or organs via Bcl-2 activation [25].

*Angiostrongylus cantonensis*, the rat lungworm, is an important etiologic agent of eosinophilic meningitis or eosinophilic meningoencephalitis in humans [26, 27]. The infection is acquired by ingesting contaminated snails, slugs, planarians, or vegetables, and and thousands of cases of human angiostrongyliasis have been reported worldwide [28]. After invading the CNS, this parasite induces a wide range of immune responses, including eosinophil recruitment and cytokine release (IL-4, IL-5, and eotaxin) via the NF-*κ*B pathway [29]. In experimentally infected mice, blood-brain barrier (BBB) dysfunction and CSF eosinophilia always occur [30]. Chung et al. [31, 32] reported that* A*.* cantonensis* infection may cause elevation of ROS and antioxidants in the CSF of infected mice. High levels of antioxidants produced by astrocytes may protect the surrounding neurons from oxidative stress-induced cell death by inhibiting ROS formation [33]. Moreover, astrocytes under oxidative stress are activated and secrete Shh via the PI3-K/AKT/Bcl-2 pathway [34]. In this study, we investigated the activation of the Shh signaling pathway in astrocytes of* A*.* cantonensis*-infected mice by immunohistochemistry and immunofluorescence staining. By coculturing astrocytes with living fifth-stage larvae (L5) or soluble antigens, we determined that activation of Shh signaling increases the survival rate of astrocytes through the GRP78-dependent pathway. These findings may provide valuable insight into the pathological changes during the course of cerebral angiostrongyliasis.

## 2. Materials and Methods

### 2.1. Parasite and Experimental Infection

Both SD rats and BALB/c mice were purchased from the National Laboratory Animal Center, Taipei. In our laboratory,* A*.* cantonensis* is maintained in* Biomphalaria glabrata* snails and Sprague-Dawley (SD) rats [35]. The life cycle of* A. cantonensis* was established by isolating L3 from* Achatina fulica *snail collected in Neihu, Taipei, in 1985. After infecting L3 to Sprague-Dawley (SD) rats, the first-stage larvae (L1) were recovered from the rat feces on day 50 after infection and fed to* B. glabrata *snails. To isolate L3, the infected* B. glabrata* snails were killed and the tissues were homogenized with an organization homogenizer (Cole-Parmer Instrument Co., USA) and then digested with artificial gastric juice (0.6% (w/v) pepsin, pH 2-3) at 37°C for 45 min on day 21 after infection [36]. Each mouse was inoculated with 25 larvae by stomach intubation. The infected animals were housed separately in plastic cages and provided with food and drinking water* ad libitum*. All procedures involving animals and their care were reviewed and approved by the Chang Gung University Institutional Animal Care and Use Committee (CGU12-157).

### 2.2. Preparation of* A. cantonensis* Soluble Antigens

The L5 from* A*.* cantonensis* were isolated from the brain tissues of infected mice after the mice were anesthetized with 30 *μ*L Zoletil 50 (Virbac). After washing with saline, phosphate buffered saline, and distilled water three times, the worms were disrupted by sonication in lysis buffer (8 M urea and 4% CHAPS) containing protease inhibitors (Roche Diagnostics, Basel, Switzerland) on ice for 30 times. The cell lysate was centrifuged at 10,000 ×g at 4°C for 15 min, and impurities from the total cell lysate were removed using the 2-D Cleanup Kit (GE Healthcare Bio-Science Corp., Piscataway, USA). The protein concentration in the preparations was determined with the Bio-Rad Protein Assay Kit (Bio-Rad, Hercules, CA, USA) according to the manufacturer's instructions. The soluble antigens were purified from the fifth-stage larvae of* A*.* cantonensis* with lysis buffer and the 2-D Cleanup Kit. These antigens were used to treat the astrocytes, and the cell biological changes were then observed.

### 2.3. Cell Culture

Mouse astrocytes (CRL-2535) were purchased from the American Type Culture Collection. Dulbecco's modified Eagle's medium supplemented with 10% fetal bovine serum and 100 U/mL penicillin/streptomycin was used to maintain the cells throughout the study. The cells were seeded onto poly-L-lysine coated culture plates at 0.25 × 10^6^ cells/cm^2^ at 37°C under 5% CO_2_. After culturing for seven days, the cells grew to a confluent layer of 1-2 × 10^4^ cells/cm^2^. Over 95% of the cells should be detected by GFAP staining.

### 2.4. Western Blotting

The levels of the apoptosis-related proteins Bax and Bcl-2, GFAP, and Shh were measured by 12.5% SDS-PAGE. A semidry transfer unit (Bio-Rad, Hercules, CA, USA) was used to transfer the proteins from the gel to a nitrocellulose membrane at 0.04 mA for 50 min. The membrane was washed with TBS/T three times and then with blocking buffer. The primary antibodies were rabbit anti-Shh (1 : 100) (Santa Cruz Biotechnology Inc., USA), rabbit anti-Bax (1 : 500) (Santa Cruz Biotechnology Inc.), rabbit anti-Bcl-2 (1 : 500) (Santa Cruz Biotechnology Inc.), rabbit anti-GFAP (1 : 1,000) (Abcam, UK), rabbit anti-GRP78 (1 : 1000) (Sigma-Aldrich), and mouse anti-*β*-actin (1 : 5000) (Sigma-Aldrich). After incubation with these antibodies at 4°C overnight, the membranes were then washed three times before incubation with the corresponding secondary antibodies (goat anti-rabbit IgG 1 : 10,000 and rabbit anti-mouse IgG 1 : 10,000) (Sigma-Aldrich) for 45 min. The membranes were then washed with TBS/T three times and incubated with a mixture of stable peroxide solution (500 *μ*L) and enhanced solution (500 *μ*L) in the dark for 5 min. The ImageJ image analysis software (http://rsb.info.nih.gov/ij/index.html) was used to quantitate and compare the concentrations of the target proteins (GFAP, Shh, Bax, Bcl-2, and GRP78) and the control (*β*-actin).

### 2.5. ELISA

After astrocytes were treated with soluble antigen in serum-free DMEM for 0–8 h, the supernatants were collected at different time points and then centrifuged at 500 ×g for 5 min. These specimens were used to detect the concentration of Shh using a mouse-specific Shh-N ELISA kit (Sigma-Aldrich).

### 2.6. Immunohistochemistry and Immunofluorescence Staining

The brain tissues from the BALB/c mice were snap-frozen by immersion in isopentane chilled to −70°C, and the tissues were then immediately mounted in OCT medium. The samples were stored at −80°C until sectioning with a Microm OMV cryostat to 10–15 *μ*m. The frozen tissue sections were fixed in 2% (w/v) PFA (paraformaldehyde) and permeabilized in 0.5% (v/v) Triton X-100 in PBS before incubation with the purified antibodies (rabbit anti-Shh (Santa Cruz Biotechnology Inc., USA, 1 : 50) and rabbit anti-GFAP (Abcam, UK, 1 : 500)) for 2 h at room temperature or overnight at 4°C. After incubation, the sections were incubated with the appropriate secondary antibodies (DyLight 488-594-conjugate anti-rabbit IgG) (Jackson ImmunoResearch Inc., UK, 1 : 1000) for 1 h at room temperature. For nuclear counterstaining, the sections were stained with DAPI. The stained sections were then examined and photographed under a light microscope.

### 2.7. Cell Viability Assay

To determine cell survival, astrocytes (1 × 10^7^ cells/mL) were incubated with 50 mL CCK-8 (Cell Counting Kit-8) (Sigma-Aldrich) at 37°C in the dark with mild shaking for 1 h. In the presence of cells, the highly water-soluble tetrazolium salt WST-8 [2-(2-methoxy-4-nitrophenyl)-3-(4-nitrophenyl)-5-(2,4-disulfophenyl)-2H-tetrazolium, monosodium salt] produces a water-soluble formazan dye upon reduction. Cell survival is monitored by measuring the absorbance of the formazan dye at 450 nm using a spectrophotometer (Molecular Devices, USA).

### 2.8. Coculturing and Inhibition Experiments

Before coculturing the astrocytes with either L5 or soluble antigens, the cells were incubated in serum-free DMEM for 12 h. These cells were then treated with three living L5 (male and female) for 0–3 h or 500 *μ*g soluble antigens for 0–8 h. The groups were pretreated with either cyclopamine (20 *μ*M) (Sigma-Aldrich) or recombinant mouse Sonic hedgehog peptide (Shh) (3 *μ*g) (Sigma-Aldrich) for 1 h and then treated with 500 *μ*g* A. cantonensis *soluble antigens for 2 h. Cell viability was measured using the CCK-8 assay (Sigma-Aldrich).

### 2.9. Apoptosis Assay

A total of 10^6^ astrocytes were harvested and the pellet was resuspended in 1 mL of Annexin V-FITC detection kit reagent (1 mL of 1x binding buffer containing 10 *μ*L of Annexin V-FITC and 20 *μ*L of propidium iodide) (Sigma-Aldrich). These cells were incubated at room temperature in dark for 10 min. Signals were immediately analyzed and quantified using a FASCan flow cytometer (BD Biosciences, USA).

### 2.10. Statistical Analysis

Student's *t*-test was used to compare the experimental and control groups. A value of *P* < 0.05 was considered statistically significant.

## 3. Results

### 3.1. Activation of Astrocytes

**Figure 1:**
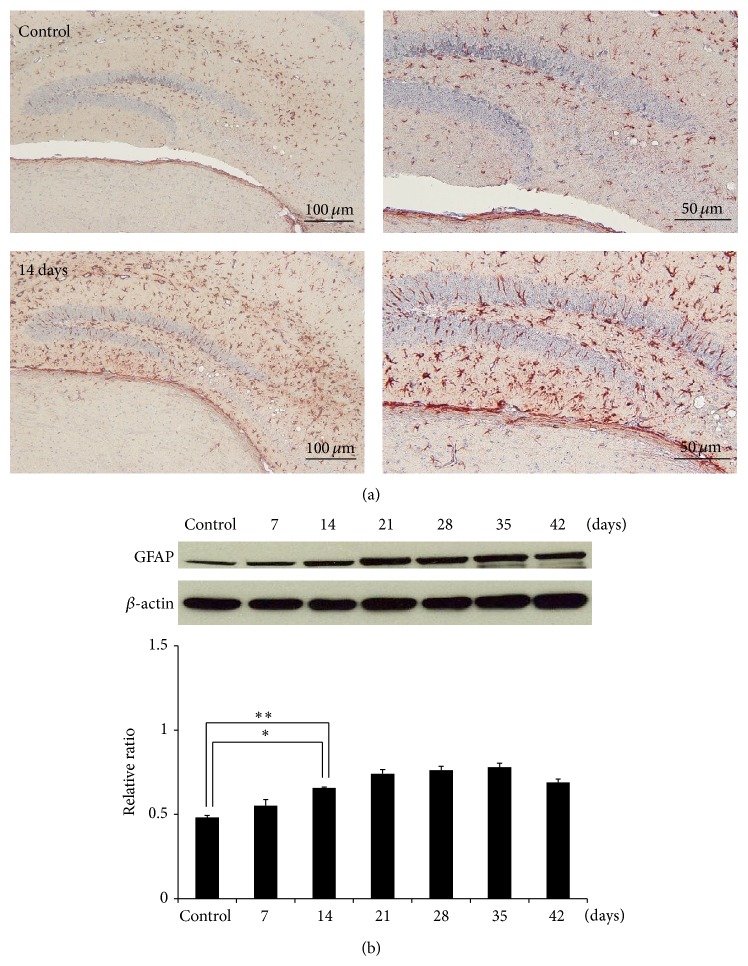
Activation of astrocytes in mice infected with* Angiostrongylus cantonensis*. (a) A significantly higher GFAP level in the hippocampus of a BALB/c mouse inoculated with 25 third-stage larvae on day 14 after infection was determined by staining with rabbit anti-GFAP. (b) Results from infected mice from day 7 to day 42 after infection were confirmed by Western blotting (*n* = 3, ^∗^ *P* < 0.05, and ^∗∗^ *P* < 0.01).

The presence of activated astrocytes in the brain sections was detected by immunohistochemistry. GFAP expression in the astrocytes was significantly higher in the hippocampus as determined by staining with rabbit anti-GFAP on day 14 after infection (Figure 1(a)). Western blotting also showed that GFAP expression was significantly higher in the infected group than the controls from day 7 (*P* < 0.05) to day 42 (*P* < 0.01) after infection (Figure 1(b)).

### 3.2. Activation of the Shh Pathway and Expression of Shh

**Figure 2:**
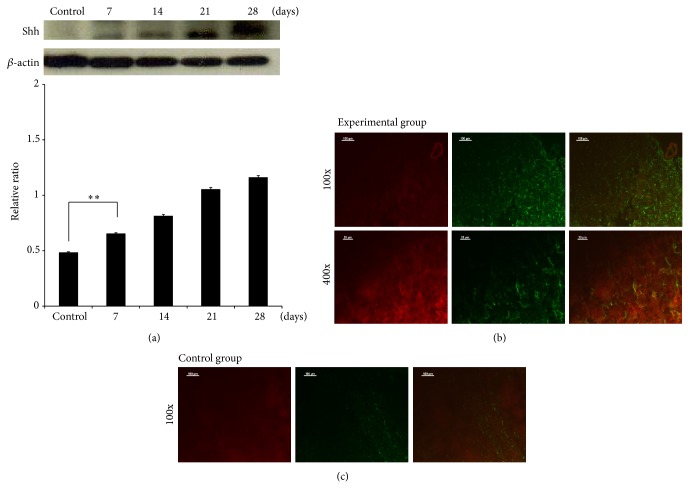
Elevation of Shh expression in mice infected with* Angiostrongylus cantonensis*. (a) Western blotting showing significant increases in the levels of Shh from day 7 to day 28 after infection (*n* = 3, ^∗^ *P* < 0.01). (b) An increased Shh expression was demonstrated by immunofluorescence staining of brain sections from an infected mouse on day 28. (c) No apparent changes were observed in a mouse without the infection on the same day (GFAP: green; Shh: red; colocalization of GFAP and Shh: yellow).

The level of Shh was increased in the infected group as determined by Western blotting. The level of the 19-kDa activated form of Shh was significantly increased beginning on day 7 after infection (Figure 2(a)). Immunofluorescence staining of brain sections from mice prepared 28 days following infection with* A*.* cantonensis* showed that the expression of GFAP and Shh was significantly increased in the cytoplasm of astrocytes (Figures 2(b) and 2(c)).

### 3.3. Culturing Astrocytes with Soluble Antigens and Worms of* A. cantonensis*

**Figure 3:**
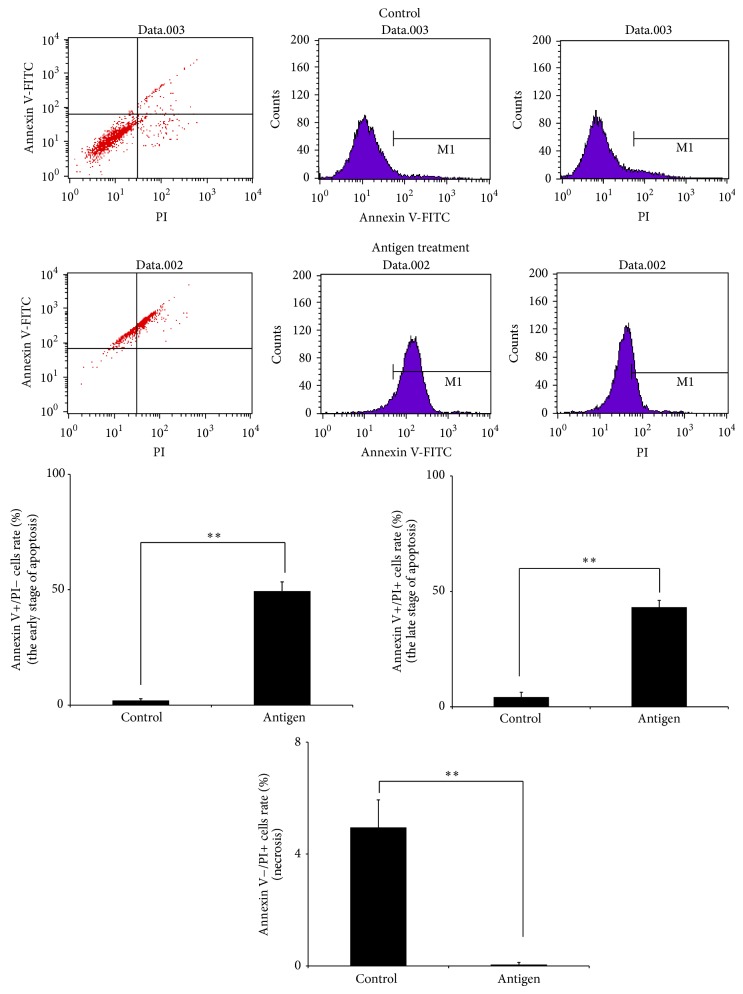
Induction of apoptosis in activated astrocytes by culturing with* Angiostrongylus cantonensis* soluble antigens. Astrocytes were treated with the 500 *μ*g antigens for 2 h. Cell apoptosis was analyzed by FASCan flow cytometer (Annexin V−/PI−: normal cells, Annexin V+/PI−: early stage apoptotic cells, Annexin V+/PI+: late stage apoptotic cells, and Annexin V−/PI+: necrosis cells) (*n* = 3, ^∗∗^ *P* < 0.01).

Coculturing soluble antigens or living worms of* A*.* cantonensis* with astrocytes was performed to determine the induction of Shh release and apoptosis occurred in activated astrocytes. To assay apoptosis in astrocytes treated with* A. cantonensis* soluble antigens, changes in Annexin V-FITC and propidium iodide were analyzed (Figure 3). In the control group, only 2.07% were early stage apoptotic cells (Annexin V+/PI− cells) whereas the percentage in the cells treated with 500 *µ*g/ml antigens was 49.44%. In addition, 4.16% in the control group were late stage apoptotic cells (Annexin V+/PI+ cells) while the corresponding percentage in the experimental group was 43.6%. However,* A. cantonensis* antigens could not induce necrosis in astrocytes (Annexin V−/PI+) (4.95% in control group versus 0.5% in experimental group). Therefore, the apoptosis of astrocytes was induced in* A. cantonensis* infection.

**Figure 4:**
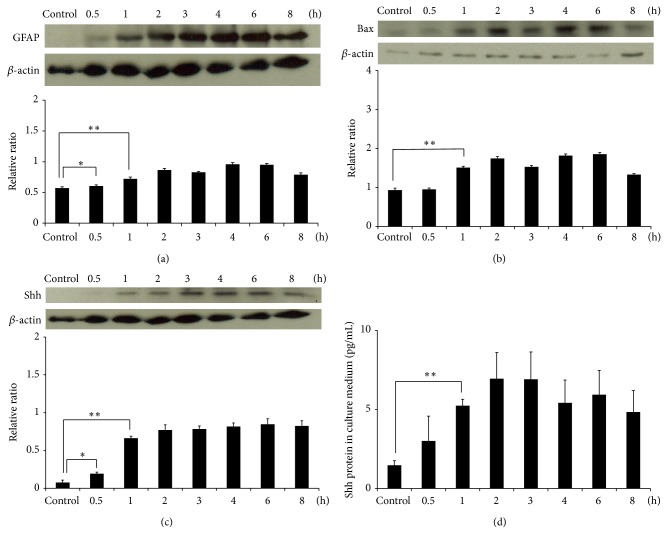
Induction of Shh release and apoptosis in activated astrocytes by culturing with* Angiostrongylus cantonensis* soluble antigens. Astrocytes were treated with the 500 *μ*g antigens for 0.5–8 h. Western blotting showed significant increases in the expressions of (a) GFAP, (b) Bax, and (c) Shh in astrocytes after 1 h. (d) The elevation of Shh concentration in the culture medium was confirmed by ELISA (*n* = 3, ^∗^ *P* < 0.05, and ^∗∗^ *P* < 0.01).

**Figure 5:**
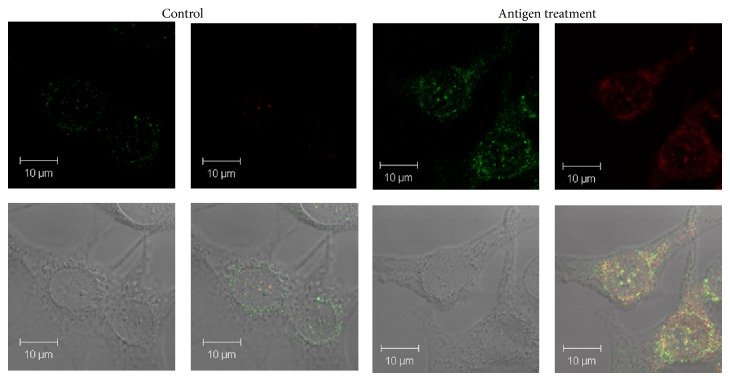
Expression of Shh and GFAP in activated astrocytes by culturing with* Angiostrongylus cantonensis* soluble antigens. Astrocytes were treated with the 500 *μ*g antigens for 2 h. Increases in the levels of Shh and GFAP expressions were detected by immunofluorescence staining of normal and antigen treated astrocytes. Magnification is 1000x (GFAP: green; Shh: red; colocalization of GFAP and Shh: yellow).

**Figure 6:**
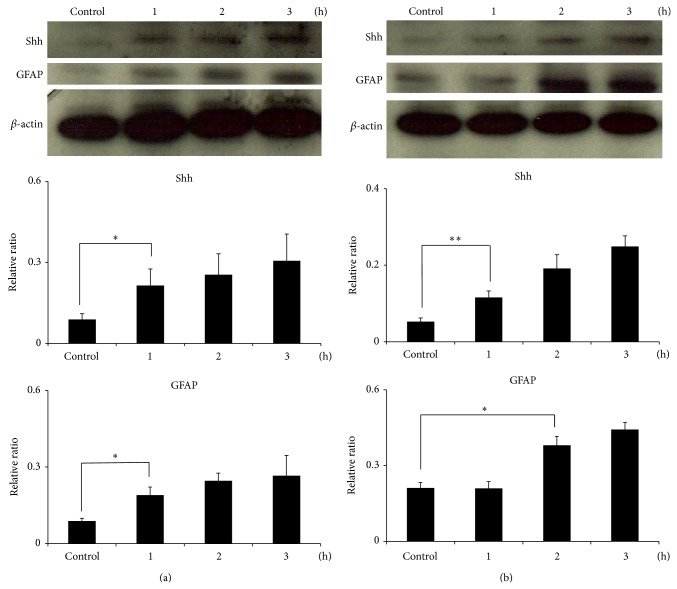
Induction of astrocyte activation and Shh expression by coculturing astrocytes with* Angiostrongylus cantonensis*. (a) Male worms. (b) Female worms. The levels of the GFAP and Shh were significantly increased after 2 h (*n* = 3, ^∗^ *P* < 0.05, and ^∗∗^ *P* < 0.01).

Western blotting showed that the levels of GFAP, Bax, and Shh in astrocytes were significantly increased after culturing with 500 *μ*g* A*.* cantonensis* soluble antigens for 1 h (*P* < 0.01) (Figures 4(a)–4(c)). The level of Shh was also determined by ELISA and was significantly elevated in the culture medium after 1 h (*P* < 0.01) (Figure 4(d)). Immunofluorescence staining also detected the location of Shh and GFAP in the cytoplasm of astrocytes treated with* A*.* cantonensis* soluble antigens (Figure 5). In addition, coculturing the astrocytes with male (Figure 6(a)) or female (Figure 6(b))* A. cantonensis* worms also showed increasing levels of GFAP and Shh after 2 h (*P* < 0.01).

### 3.4. Survival Enhancement via the GRP78-Dependent Pathway

**Figure 7:**
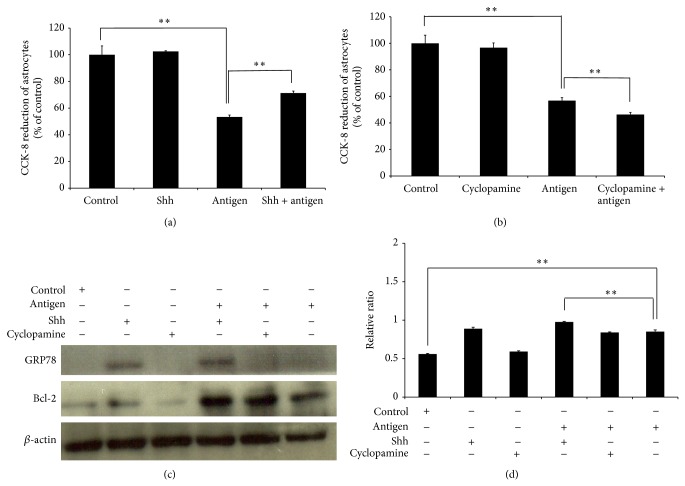
Activation of the Shh signaling pathway in astrocytes via a GRP78-dependent pathway. Astrocytes were pretreated with (a) Shh (3 *μ*g) or (b) cyclopamine (20 *μ*M) for 1 h and then treated with soluble* Angiostrongylus cantonensis* antigens (500 *μ*g) for 2 h. Cell viability was measured using a CCK-8 assay. (c) Detection of GRP78 and Bcl-2 expression during* A*.* cantonensis* infection. The cultured astrocytes were pretreated with Shh (3 *μ*g) or cyclopamine (20 *μ*M) for 1 h and then treated with the antigens for 2 h. (d) The expression of GRP78 was detected with the ImageJ software (*n* = 3, ^∗^ *P* < 0.05, and ^∗∗^ *P* < 0.01).

A CCK-8 assay was employed to determine the survival of astrocytes after coculturing experiments with soluble antigens or treatment with a signaling inhibitor. The survival rates of astrocytes treated with the soluble antigens and Shh were significantly higher than those treated with the soluble antigens only (*P* < 0.01) (Figure 7(a)). However, the survival rates of astrocytes treated with cyclopamine, a Shh pathway signaling inhibitor, were lower than those treated with the soluble antigens only (*P* < 0.01) (Figure 7(b)). The expression of GRP78 was higher in astrocytes pretreated with Shh (*P* < 0.01). However, the expression of GRP78 and Bcl-2 was also higher in the cultures treated only with the soluble antigens (*P* < 0.01). Moreover, the expression of GRP78 and Bcl-2 was significantly higher in astrocytes pretreated with the recombinant Shh (Figures 7(c) and 7(d)). These findings suggest that the expression of Shh may inhibit cell death by activating Bcl-2 through a GRP78-dependent pathway.

## 4. Discussion

The rat lungworm* A*.* cantonensis* infects the central nervous system of humans and causes eosinophilic meningitis or eosinophilic meningoencephalitis [26]. After migration to the central nervous system through the blood-brain barrier, the third-stage larvae molt twice and develop into fifth-stage larvae. These larvae induce immune responses, such as eosinophil recruitment. In our previous study, we demonstrated that L5 are surrounded by inflammatory cells in the anterior cerebral fissure, hippocampus, posterior cerebral fissure, and cerebellar fissure on day 14 after infection [37]. In a previous study, high levels of cathepsin B-like cysteine proteinase 1 and hemoglobinase-type cysteine proteinase were detected in the excretory-secretory products of* A*.* cantonensis* [38]. These proteases initialize the degradation of host proteins and induce host immune responses by blood-feeding helminthes. They may be important factors that induce tissue damage in nonpermissive hosts [39].

The network of blood vessels in the brain is necessary to provide nutrients, oxygen, and hormones, as well as for removing carbon dioxide and wastes. The blood-brain barrier in these blood vessels is formed by endothelial cells and astrocytes and separates the circulating blood from the extracellular fluid in the central nervous system. The BBB only allows the diffusion of small hydrophobic molecules, such as oxygen and carbon dioxide [40]. Blood-borne pathogens, including the intracellular and extracellular parasites* Toxoplasma gondii*,* Toxocara canis*,* Trypanosoma*, and* Plasmodium* species, are able to cause blood-brain barrier dysfunction and penetrate into the central nervous system [41–44]. These parasites not only cause damage to the blood-brain barrier but also induce severe immune responses and brain damage, such as encephalitis, in the host.

After culturing with soluble antigens of* A*.* cantonensis*, levels of the apoptosis-related Bax and Bcl-2 were significantly increased in astrocytes and the activated cells were determined to be in the apoptotic state by flow cytometry (Figure 3). The elevation of these proteins suggests the occurrence of apoptosis. These findings are consistent with those observed in* A*.* cantonensis*-infected mice with meningoencephalitis [31]. Moreover, the larvae have been reported to cause severe eosinophilic meningoencephalitis in infected BALB/c mice. This pathological change in turn leads to apoptosis and death of the animals [41].

GFAP is the specific marker for astrocytes [45]. In this study, we observed a significant increase in the level of GFAP in the brains of BALB/c mice from day 7 to day 42 day after infection, suggesting that the astrocytes were activated after* A*.* cantonensis* infection. Most of the BALB/c mice infected with 25 third-stage larvae died around day 28 after infection. However, some of them may survive after this time. Therefore, detection of GFAP changes can be extended up to day 42. In in vitro experiments, we also demonstrated apoptosis and activation in astrocytes induced by* A*.* cantonensis *soluble antigens. Although* A*.* cantonensis* may cause apoptosis in some astrocytes, the remaining cells were activated by the infection and showed significant increase in the expression. Moreover, soluble antigens from* A*.* cantonensis* can also induce the activation of astrocytes, which in turn causes the significant increase in the total level of GFAP (Figure 4(a)).

In mice infected with* A*.* cantonensis*, we detected a significant increase in the expression of Shh. These specific signaling molecules include Shh, Smo, Patched, and Gli. Moreover, the expression of Shh was significantly elevated in astrocytes cultured with either living* A*.* cantonensis* worms or soluble antigens. Under oxidative stress, the level of Shh expressed in astrocytes is higher than those in neurons or fibroblasts [19]. During CNS development, Shh signaling is essential for neural tube formation [46] and brain development [5, 47]. This signaling pathway also promotes BBB integrity and immunity [14]. In brain injury or ROS generation, Shh plays a protective role by promoting cell proliferation [16, 17]. The expression of Shh in activated astrocytes may also protect neurons against oxidative stress [19].

GRP78 is an ER chaperone and may be induced under ER stress conditions. It interacts with PERK, ATF6, and IRE1 and induces the inhibition of protein synthesis and the activation of UPR genes [48]. In addition, GRP78 is an important antiapoptotic protein because it inhibits BAX and caspase-7 activation via a GRP78-dependent pathway [49] and by activating Bcl-2 expression [50]. In this study, we demonstrated the induction of the GRP78/Bcl-2 signaling pathway in mice infected with* A*.* cantonensis*. This pathway is significantly increased during the overexpression of Shh signaling. These findings suggest that the expression of Shh via a GRP78-dependent pathway may significantly increase the survival of astrocytes. In conclusion, the activation of the Shh signaling pathway may protect astrocytes by activating antiapoptotic proteins, such as Bcl-2 and GRP78, after* A*.* cantonensis* infection.

In the present study, the role of the Shh signaling pathway in astrocytes was investigated. We used the specific inhibitor cyclopamine to inhibit the Shh pathway and cause cell death in the presence of soluble antigens. In addition, exogenous Shh increased the survival of astrocytes. These findings indicate that the Shh signaling pathway plays a protective role to astrocytes in BALB/c mice infected with* A. cantonensis*. Moreover, the activation of the Shh signaling pathway increased the expression of GRP78 in infected mice.
